# Influence of Precision of Emission Characteristic Parameters on Model Prediction Error of VOCs/Formaldehyde from Dry Building Material

**DOI:** 10.1371/journal.pone.0080736

**Published:** 2013-12-03

**Authors:** Wenjuan Wei, Jianyin Xiong, Yinping Zhang

**Affiliations:** 1 Department of Building Science, Tsinghua University, Beijing, China; 2 School of Mechanical Engineering, Beijing Institute of Technology, Beijing, China; The Ohio State University, United States of America

## Abstract

Mass transfer models are useful in predicting the emissions of volatile organic compounds (VOCs) and formaldehyde from building materials in indoor environments. They are also useful for human exposure evaluation and in sustainable building design. The measurement errors in the emission characteristic parameters in these mass transfer models, i.e., the initial emittable concentration (*C*_0_), the diffusion coefficient (*D*), and the partition coefficient (*K*), can result in errors in predicting indoor VOC and formaldehyde concentrations. These errors have not yet been quantitatively well analyzed in the literature. This paper addresses this by using modelling to assess these errors for some typical building conditions. The error in *C*_0_, as measured in environmental chambers and applied to a reference living room in Beijing, has the largest influence on the model prediction error in indoor VOC and formaldehyde concentration, while the error in *K* has the least effect. A correlation between the errors in *D*, *K*, and *C*_0_ and the error in the indoor VOC and formaldehyde concentration prediction is then derived for engineering applications. In addition, the influence of temperature on the model prediction of emissions is investigated. It shows the impact of temperature fluctuations on the prediction errors in indoor VOC and formaldehyde concentrations to be less than 7% at 23±0.5°C and less than 30% at 23±2°C.

## Introduction

Chemical pollutant emissions from building materials in indoor environments can result in adverse health effects on indoor occupants [1], [2]. Analytical emission models predicting VOC/formaldehyde emissions provide an efficient way to evaluate the short and long term emissions of VOCs/formaldehyde in various practical environmental conditions as compared to chamber studies of emissions. The accurate prediction of indoor VOC/formaldehyde concentrations is the basis for the evaluation and subsequent control of indoor VOC/formaldehyde exposure. A series of mass transfer models have been developed based on different initial or boundary conditions to predict the emissions of VOCs/formaldehyde from building materials [3], [4]. The predicted emission rates of VOCs/formaldehyde using different emission models for a single layer homogeneous building material differ mainly during the initial stage when the dimensionless mass transfer time, Fourier number (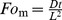, where, *D* is the diffusion coefficient, *t* is time, and *L* is the thickness of the building material), is less than 10^−4^, but are similar when used for long term prediction [5]–[7].

Three emission characteristic parameters need to be measured in advance for model predictions: the diffusion coefficient of VOCs/formaldehyde in building material, *D* (m^2^/s); the material/air phase partition coefficient, *K* (−); and the initial emittable VOC/formaldehyde concentration, *C*_0_ (µg/m^3^).

Several experimental methods have been developed to determine the emission characteristic parameters, for example, the cup method, the CLIMPAQ method, the two chamber method, the microbalance method, and the porosity test method for *D*; the multi-sorption equilibrium regression method, the multi-emission/flush regression method, and the variable volume loading (VVL) method for *K*; and, the CM-FBD (cryogenic milling - fluidized bed desorption) method, the multi-flushing extraction method, and the C-history method for *C*_0_ [3]. Since *D*, *K*, and *C*_0_ are the input characteristic parameters for model predictions of VOC/formaldehyde emission, we need to know what effect any measurement error in these parameters will have on predicting indoor VOC/formaldehyde concentrations.

Some studies show that measurement errors in *D*, *K*, and *C*_0_ can result in the error in model prediction of chamber VOC/formaldehyde concentration [8]. And conversely, the measurement error in chamber VOC/formaldehyde concentration can result in errors when determining *D*, *K*, and *C*_0_ [7]. The relationship between the measurement errors in *D*, *K*, and *C*_0_ and the model prediction error in chamber VOC/formaldehyde concentration needs to be addressed more quantitatively. The purpose of this paper is to quantitatively analyze the relationship between the measurement errors in the VOC/formaldehyde emission characteristic parameters of building materials and the model prediction error in indoor concentration.

### Measurement Errors in VOC/Formaldehyde Emission Characteristic Parameters

The measurement errors in VOC/formaldehyde emission characteristic parameters arise firstly from systematic errors in the measurement system, and secondly from random operating errors. Analysis of the measurement errors in the VOC/formaldehyde emission characteristic parameters can be divided into three categories: measurement errors in one laboratory employing one evaluation method; comparison of different evaluation methods; and measurement errors in inter-laboratory studies.

### Measurement Errors in one Laboratory Employing One Evaluation Method

**Figure 1:**
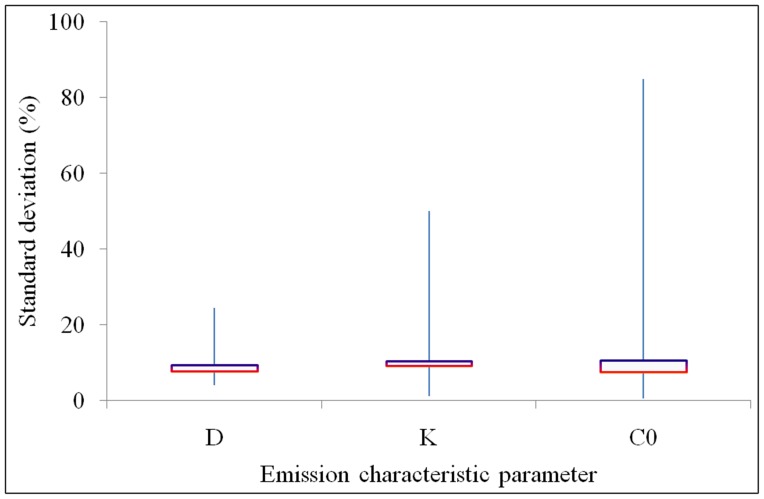
Measurement errors in *D*, *K*, and *C*_0_ within one laboratory employing one evaluation method.

Measurement errors in one laboratory employing one evaluation method indicate the random operating errors between replicated measurements. In reviewing the literature published before 2013 for measuring *D*, *K*, and *C*_0_ we found that the diffusion coefficients range from 10^−14^ m^2^/s to 10^−10^ m^2^/s, the partition coefficients from 10^1^ (−) to 10^5^ (−), and the initial emittable concentrations from 10^3^ µg/m^3^ to 10^7^ µg/m^3^ in materials such as medium density board and vinyl flooring for VOCs and formaldehyde [9]–[16]. Measurement errors in *D*, *K*, and *C*_0_ are shown in Figure 1. The error bars for *D*, *K*, and *C*_0_ from 0% to 100% represent the full range of standard deviations for the measurement data found in the literature. The squares in the centres of the error bars for *D*, *K*, and *C*_0_ represent the median value of the standard deviations as the bottom horizontal red lines, and the mean value of the standard deviations as the top horizontal purple lines. The median values of the standard deviations for *D*, *K*, and *C*_0_ are smaller than the mean values, probably because the errors in a few measurements are much higher than the others. The median values of the standard deviations of the measurement data are 8% for *D*, 9% for *K*, and 7% for *C*_0_. The mean values of the standard deviations of the measurement data are 9% for *D*, 10% for *K*, and 11% for *C*_0_.

### Comparison of Different Evaluation Methods

**Figure 2:**
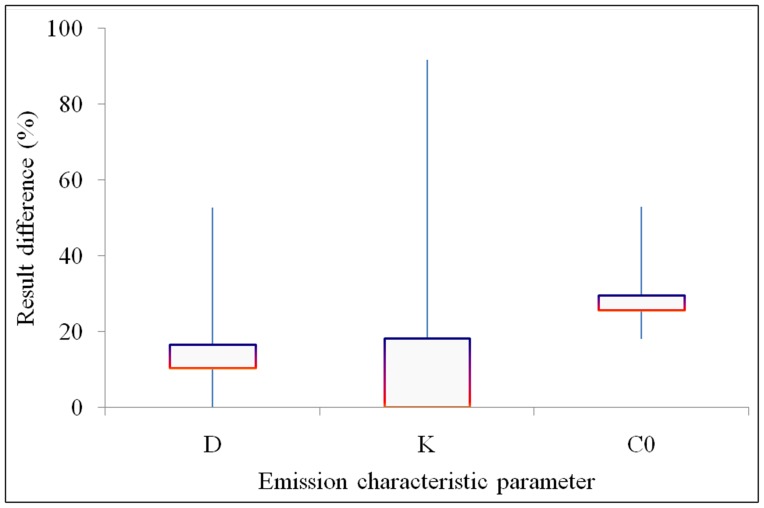
Comparison of different methods for determing *D*, *K*, and *C*_0_.

Since there are various methods to measure *D*, *K*, and *C*_0_ of building materials, comparing the methods when measuring replicated materials will give an indication of the difference between methods. The difference between methods occurs in testing protocols [10], [17] or data analysis protocols [7]. The measurement results for *D*, *K*, and *C*_0_ using different methods are shown in Figure 2. The error bars for *D*, *K*, and *C*_0_ from 10% to 160% represent the full range of differences for the measurement data that was found in the literature. The squares in the centres of the error bars for *D*, *K*, and *C*_0_ represent the median value of the differences between methods as the horizontal red lines, and the mean value of the differences between methods as the horizontal purple lines. The median values of the differences between methods are 11% for *D*, less than 1% for *K*, and 26% for *C*_0_. The mean values of the differences between methods are 17% for *D*, 18% for *K*, and 30% for *C*_0_. As a result, the measurement errors for *D*, *K*, and *C*_0_ range from 10% to 30%.

### Measurement Errors in Inter-laboratory Studies

**Figure 3:**
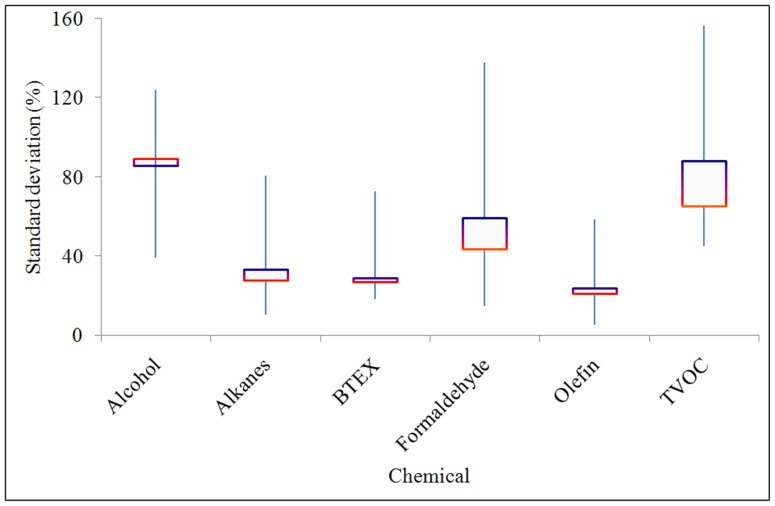
Inter-laboratory studies for measuring VOC/formaldehyde concentrations in chamber air.

Tests to determine VOC/formaldehyde emissions from building materials are usually performed in environmental chambers, as are the tests to determine the emission characteristic parameters. The VOC/formaldehyde molecules in the test material diffuse into the air in the chamber, which can then be sampled and analyzed by using instruments such as a GC/MS, and HPLC, thereby measuring the concentration of VOCs/formaldehyde in the air in the chamber. Inter-laboratory studies were made to address the deviations of results between laboratories in measuring the VOC/formaldehyde concentrations in chamber air by using reference emission samples [18]–[22]. The standard deviations of results between laboratories in measuring the concentrations of alcohol, alkanes, BTEX, formaldehyde, olefin, and TVOC in the chamber air are shown in Figure 3. The error bars in the figure show the full range of the standard deviations in the inter-laboratory studies for measuring VOC/formaldehyde concentrations in chamber air. The squares in the centres of the error bars represent the median value of the standard deviations of the inter-laboratory comparison results as the horizontal red lines, and the mean value of the standard deviations of the inter-laboratory comparison results as the horizontal purple lines. The median values of the standard deviations of the inter-laboratory comparisons are 89% for alcohol, 28% for alkanes, 27% for BTEX, 44% for formaldehyde, 21% for olefin, and 65% for TVOC. The mean values of the standard deviations of the inter-laboratory comparisons are 86% for alcohol, 33% for alkanes, 29% for BTEX, 59% for formaldehyde, 24% for olefin, and 88% for TVOC.

### Possible Reasons for the Errors in VOC/Formaldehyde Emission Measurements

Errors in VOC/formaldehyde emission measurements result from three possible causes. One is the difference between the measurement methods used to determine the emission characteristic parameters, especially the initial emittable VOC/formaldehyde concentration in building materials. Another is the performance of the measurement systems, e.g., the temperature control accuracy, air leakage into the environmental chamber, and the accuracy of the air sampling volume. The measurement errors caused by the performances of these systems can be systematic errors and random errors, and will generally differ between laboratories. The third is the performance of the chemical analysis instruments, e.g., GC/MS for analyzing VOCs and HPLC for analyzing formaldehyde. This error can be minimized by regularly calibrating the instruments.

## Methods

### Mass Transfer Model to Predict VOC/Formaldehyde Emissions from Building Materials

After decades of study, dozens of emission models have been developed to predict the emission of VOCs/formaldehyde from dry building material. Among those models, Deng’s model [6] provides a fully analytical solution for predicting VOC/formaldehyde emissions from a single layer of homogeneous material under ventilated conditions when the chamber inlet VOC/formaldehyde concentration and the initial VOC/formaldehyde concentration in the chamber air are zero. More generous models such as Xu’s model and Wang’s model are semi-analytical solutions that require a finite difference method to solve [5], [23]. Since Deng’s model gives results consistent with experimental measurements, it has been widely used by many researchers [3], [7]. As it is good for predicting building material emissions under common practical conditions, we have used it to predict VOC/formaldehyde emissions from building materials in this study. The VOC/formaldehyde concentration in the chamber air using Deng’s model can be predicted as:
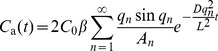
(1)where, *C*_a_(t) is the predicted VOC/formaldehyde concentration in the chamber (µg/m^3^); *L* is the thickness of the material (m); and *t* is the time for prediction (s).

With
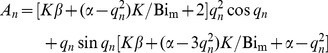
(2)

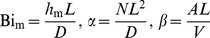
(3)where, *A* is the emission area of the material (m^2^); *V* is the volume of the chamber (m^3^); *N* is the air change rate in the chamber (h^−1^); and *h*_m_ is the convective mass transfer coefficient in the chamber (m/s). *q_n_* is the positive root of the following equation:
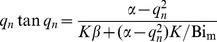
(4)


### Error analysis of Model Prediction in a Ventilated Chamber and a Reference Living Room

Experimental data from environmental chambers are available from previous chamber studies reported in the literature, e.g., the conditions of the chamber and the parameters of the building material found in Yao [24], Table 1. Formaldehyde concentration in the air in a 30 m^3^ ventilated chamber loaded with board furniture was measured for a period of a week. The formaldehyde concentration in the chamber air was measured by INNOVA-1312 as well as being predicted using Deng’s model. As discussed in previous sections, the measurement errors in *D*, *K*, and *C*_0_ in statistical analysis are in the range from 10% to 30%. Therefore, reasonable errors ±20% in the measurement data of *D*, *K*, and *C*_0_ were then added for model predictions of formaldehyde concentrations in the chamber.

**Table 1:** Parameters of the chamber and the building material.

Chamber		Building material	
Temperature (°C)	23±0.5	Material	Board furniture
Relative humidity	50±5	Emission area (m^2^)	16.29
Air change rate (h^−1^)	1	Thickness (m)	0.019
Volume (m^3^)	30	Diffusion coefficient (m^2^/s)	3.13×10^−10^
Convective mass transfer coefficient (m/s) [5]	1.20×10^−3^	Partition coefficient (−)	482
		Initial emittable concentration (µg/m^3^)	5.10×10^5^

One of the main applications of the emission models is to predict VOC/formaldehyde emissions from furniture in the indoor environment, e.g., office room, living room, bedroom, which can be a useful tool for indoor VOC/formaldehyde source control or indoor decorating guidelines for chemical pollution. The model prediction in a real furnished room requires the input data of the VOC/formaldehyde emission characteristic parameters (*D*, *K*, and *C*_0_) of the furniture and the information about the room, such as room volume, furniture loading factor, ventilation etc. [25]. Since the measured parameters (*D*, *K*, and *C*_0_) always have errors, the influence of the measurement errors in *D*, *K*, and *C*_0_ on the model prediction error in indoor VOC/formaldehyde concentration needs to be studied.

The information of the reference living room (obtained by Yao in 2011 from a survey of 1500 homes in Beijing) is used in this error analysis for model prediction of emissions. The information of the reference living room in Beijing includes the volume, furniture loading factor, ventilation etc. The information of the reference living room and the emission characteristic parameters of the furniture are given in Table 2. Formaldehyde is selected as the chemical pollutant. Deng’s model is used for the prediction of the indoor formaldehyde concentration.

**Table 2:** Parameters of the reference living room and the furniture.

Reference living room		Furniture	
Volume (m^3^)	57.2	Mean thickness of board (m)	0.019
Loading factor (m^2^/m^3^)	0.42	Diffusion coefficient (m^2^/s)	3.13×10^−10^
Air change rate (h^−1^)	1	Partition coefficient (−)	482
Convective mass transfer coefficient (m/s)	1.20×10^−3^	Initial emittable concentration (µg/m^3^)	5.10×10^5^

## Results and Discussion

### Error analysis of Model Prediction in a Ventilated Chamber

**Figure 4:**
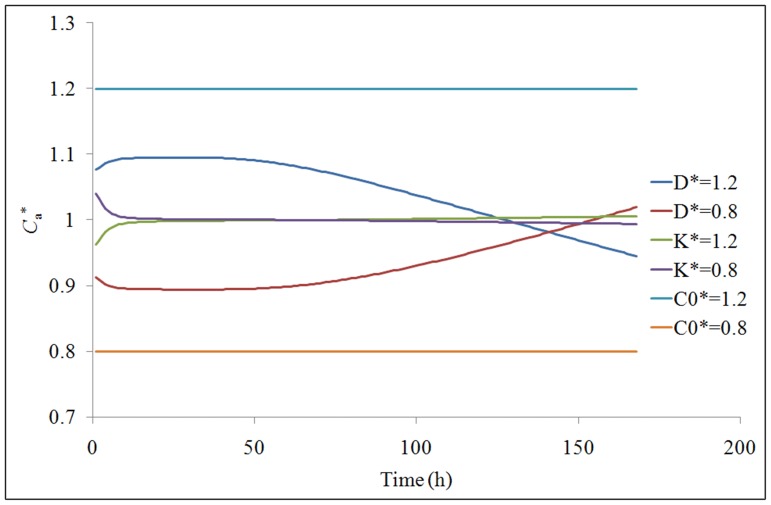
Influence of the measurement errors in *D*, *K*, and *C*_0_ on the model prediction errors in *C*_a_ (single variable).

The influence of the measurement errors in *D*, *K*, and *C*_0_ on the model prediction errors in *C*_a_ is shown in Figure 4. The normalized *D*, *K*, and *C*_0_ (represented as *D* ^*^, *K* ^*^, and *C*_0_^*^) are the ratio of *D*, *K*, and *C*_0_ with given errors (±20%), to the measurement data of *D*, *K*, and *C*_0_. The normalized *C*_a_ (represented as *C*_a_^*^) is the ratio of the model prediction value of *C*_a_ using *D*, *K*, and *C*_0_ with given errors to the model prediction value of *C*_a_ using the measurement data of *D*, *K*, and *C*_0_. The values of *C*_a_^*^ are the same as the input values of *C*_0_^*^ indicating the errors in *C*_0_ transferred directly into the model prediction errors in *C*_a_. The influence of the errors in *D* and *K* on the model prediction errors in *C*_a,_ changes over time. A positive error in *D* results in a positive error in *C*_a_ during the initial emission period. In contrast, a positive error in *K* results in a negative error in *C*_a_ during the initial emission period. A 20% error in *D* results in a maximum of 10% error in *C*_a_. A 20% error in *K* results in a maximum of 4% error in *C*_a_. Therefore, the error in *C*_0_ has the greatest influence on the model prediction error in *C*_a_ while the error in *K* has the least influence on the model prediction error in *C*_a_.

**Figure 5:**
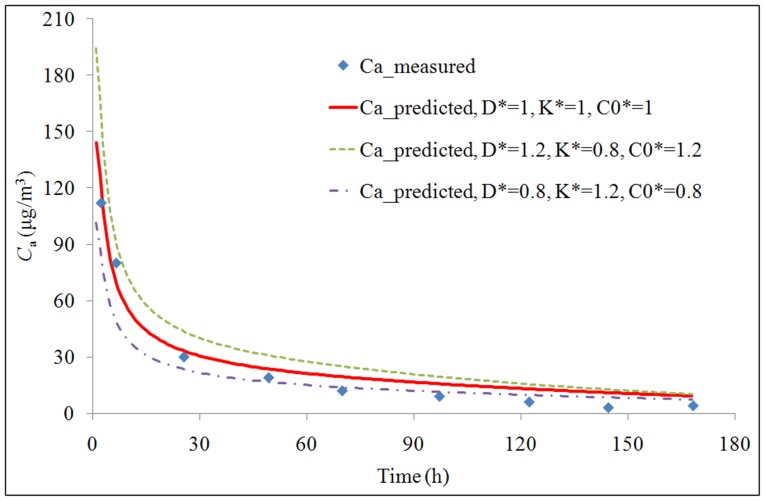
Measurement data and model prediction of formaldehyde concentrations in a ventilated chamber.

The measurement data and the model prediction of formaldehyde concentrations in the ventilated chamber (*C*_a_) are shown in Figure 5. The *R* ^2^ value between the measurement data and the model prediction is 0.96. As analyzed before, positive errors in *C*_0_ and *D* and negative error in *K* result in positive error in *C*_a_. The maximum positive error in *C*_a_ exists when a 20% error exists in *C*_0_ and *D*, and a −20% error exists in *K*. The maximum negative error in *C*_a_ exists when a −20% error exists in *C*_0_ and *D*, and a 20% error exists in *K*. The maximum positive and negative errors in *C*_a_ are 35% and −29%, respectively. Comparing the *C*_a_ curve with positive error to the original *C*_a_, the R-value and p-value are 0.99 and 0.009, respectively. Comparing the *C*_a_ curve with negative error to the original *C*_a_, the R-value and p-value are 0.99 and 0.001, respectively.

### Error Analysis of Model Prediction in a Reference Living Room

The error transfer in the reference living room is the same as in the ventilated chamber. The maximum positive error in *C*_a_ is 35% when a 20% error exists in *C*_0_ and *D*, and a −20% error exists in *K*. The maximum negative error in *C*_a_ is −29% when a −20% error exists in *C*_0_ and *D*, and a 20% error exists in *K*.

**Figure 6:**
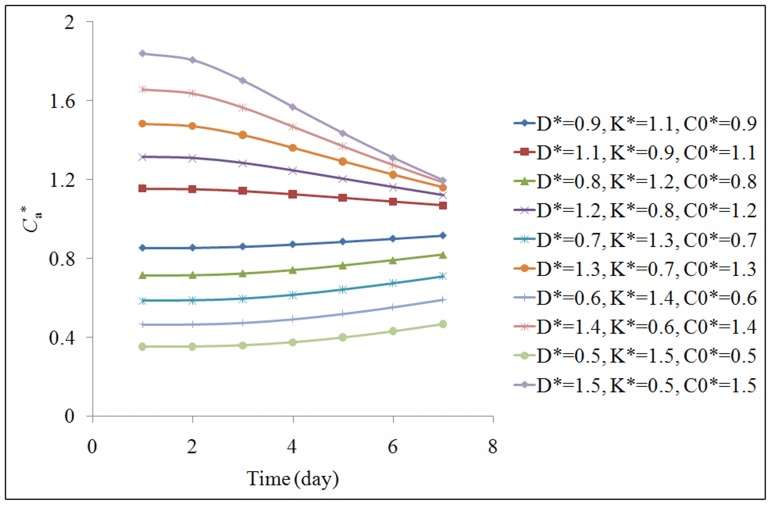
Influence of the measurement errors in *D*, *K*, and *C*_0_ on the model prediction errors in *C*_a_ (multi variables).

A series of error analyses are shown in Figure 6. The errors in *D*, *K*, and *C*_0_ are set in the range from ±10% to ±50% of their mean value. The errors in *D*, *K*, and *C*_0_ can all result in the errors in the predicted *C*_a_. The additive effect magnifies the maximum errors of *C*_a_, which are in the range of ±15% when the errors in the input *D*, *K*, and *C*_0_ are in the range of ±10%. However, as time passes, the errors in the predicted *C*_a_ tend to converge.

To address the correlation between *D* ^*^, *K* ^*^, *C*_0_^*^, and *C*_a_^*^, the errors in *D*, *K*, and *C*_0_ are randomly set by computer from ±10% to ±50% and the error in *C*_a_ is calculated using Deng’s model. The series of the error analysis data were studied using the Lervenberg-Marquardt algorithm for regression to obtain the correlation between *D* ^*^, *K* ^*^, *C*_0_^*^ and *C*_a_^*^. The fitted correlation for *D* ^*^, *K* ^*^, *C*_0_^*^, and *C*_a_^*^ is written as:
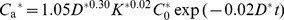
(5)


The *R* ^2^ value between the model prediction value of *C*_a_^*^ and the correlation calculated value of *C*_a_^*^ is 0.93 when *D* is of the magnitude of 10^−10^ m^2^/s, *K* is of the magnitude of 10^2^ (−), and *C*_0_ is of the magnitude of 10^5^ µg/m^3^.

### Influence of Temperature on Model Prediction of Emissions

Temperature fluctuation is a common phenomenon in reality when measuring VOC/formaldehyde emissions from building materials in environmental chambers and in households that use air conditioning. There are standard guidelines for controlling temperature when measuring VOC/formaldehyde emissions from building materials in environmental chambers e.g., ASTM D6670 specifies that the temperature be 23±0.5°C while the Chinese GB 18584 has a temperature requirement of 23±2°C [26], [27].

Temperature is an important environmental factor that has a great influence on the emission characteristic parameters of building materials. The experimental correlations between temperature and the emission parameters (*D*, *K*, and *C*_0_) in previous studies for formaldehyde in medium density board are summarized as:
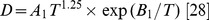
(6)

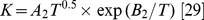
(7)

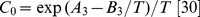
(8)where, *T* is the temperature (K); *A*_1_, *A*_2_, *A*_3_, *B*_1_, *B*_2_, *B*_3_ are the constants independent of temperature. Based on equations (6)–(8), the following relationships can be derived:



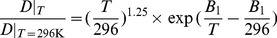
(9)

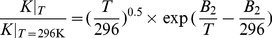
(10)

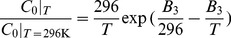
(11)where, *B*_1_ = −8032, *B*_2_ = 6741, and *B*_3_ = 7030.

The calculation of the temperature effect was performed in the reference living room in Beijing using Deng’s model. The temperature and the emission parameters (*D*, *K*, and *C*_0_) at each temperature are calculated using the experimental correlations (9) to (11) and are listed in Table 3. The temperature fluctuation from 295.5 K to 296.5 K can result in errors in *D* from −5% to 5%, in *K* from 4% to −4%, and in *C*_0_ from −4% to 4%, respectively. A temperature fluctuation from 294 K to 298 K can result in errors in *D* from −18% to 21%, in *K* from 16% to −14%, and in *C*_0_ from −14% to 16%, respectively.

**Table 3:** Parameters for temperature influence study.

Temperature (K)	Diffusion coefficient (m^2^/s)	Partition coefficient (−)	Initial emittable concentration (µg/m^3^)
294	2.58×10^−10^	561	4.37×10^5^
295.5	2.98×10^−10^	501	4.91×10^5^
296	3.13×10^−10^	482	5.10×10^5^
296.5	3.28×10^−10^	464	5.30×10^5^
298	3.79×10^−10^	415	5.94×10^5^

**Figure 7:**
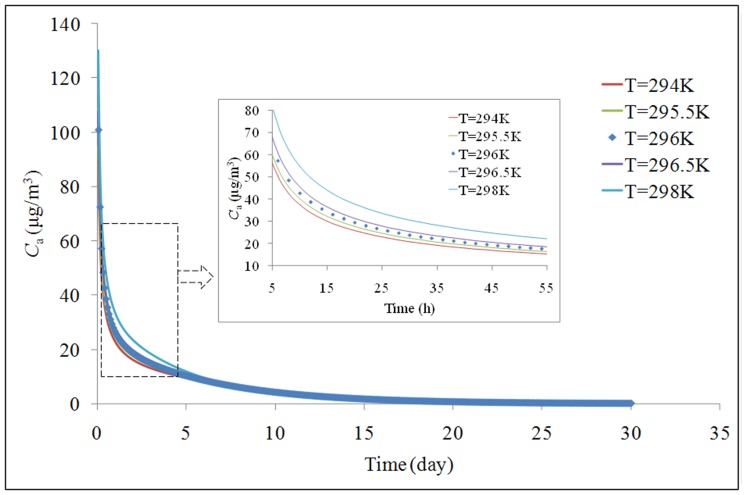
Temperature influence on model prediction of emissions.

A simulation of the indoor formaldehyde concentration in the reference living room over 1 month is shown in Figure 7. The concentration decreases from 101 µg/m^3^ to below 5 µg/m^3^. The temperature fluctuation range of 22.5°C to 23.5°C can result in a maximum error range in *C*_a_ of −6% to 7%. The temperature fluctuation range from 21°C to 25°C can result in a maximum error range in *C*_a_ of −10% to 29%. The values of *C*_a_ obtained under temperature fluctuation are compared with the value of *C*_a_ at 23°C. The p-values for the comparisons are much less than 0.01. In general, an increase in temperature results in positive errors in *C*_a_, and a decrease in temperature results in negative errors in *C*_a_.

Model predictions of VOC/formaldehyde emissions from building materials make some assumptions to simplify the model, e.g., homogenous material, one dimension mass transfer and no chemical reactions. However, emissions of VOC/formaldehyde from building materials in reality are always much more complex. Environmental conditions such as temperature fluctuations, relative humidity, and air speed, can cause fluctuation of the VOC/formaldehyde concentration. Chemical reactions for active VOCs and formaldehyde in building materials and the air sometimes occur, e.g., in the presence of ozone. However, model predictions provide ideal evaluations of VOC/formaldehyde concentrations in the indoor environment, and can agree well with the experimental data from well-performed environmental chamber tests [8], [31], [32].

## Conclusions

This study investigates the influence of the measurement accuracy of the emission characteristic parameters of building materials on model prediction error when using a model to predict emissions in environmental chambers and in a reference living room in Beijing. A general correlation between the errors in the emission parameters (*D*, *K*, and *C*_0_), and the error in the model prediction value of VOC/formaldehyde concentration (*C*_a_) in the air in indoor environment is derived. The error in *C*_0_ has the largest and linear influence on the error in *C*_a_, while the error in *K* has the least influence. The largest error in *C*_a_ always appears in the initial emission period and tends to converge thereafter. Temperature is an important environmental factor, the control accuracy of which can affect the emission parameters of *D*, *K*, and *C*_0_, and thus influences the calculation using mass transfer models and results in the errors in predictions of *C*_a_. Since temperature fluctuations can increase or decrease *D* and *C*_0_ at the same time, the additive effect always enhances the errors in *C*_a_ during the initial emission period.

This study provides statistical results summarizing the measurement accuracy of emission key parameters in previous studies, and addresses the influence of measurement errors in emission characteristic parameters on model prediction of indoor VOC/formaldehyde concentrations. It is shown in this statistical study that the measurement errors in the emission characteristic parameters can reasonably result in 10% to 30% prediction errors in indoor VOC/formaldehyde concentrations. It might be helpful for researchers or engineers who do simulations of indoor air chemical pollutants to carefully select the emission characteristic parameters for model predictions.
